# Response to Letter to the Editor

**DOI:** 10.5041/RMMJ.10553

**Published:** 2025-07-31

**Authors:** Poonam Joshi, Manasi Bavaskar

**Affiliations:** 1 Department of Head and Neck Surgical Oncology, Tata Memorial Centre, Advanced Centre for Treatment, Research & Education in Cancer, Mumbai, Maharashtra, India; 2 Department of Surgical Oncology, MPCT Hospital, Mumbai, Maharashtra, India

**Keywords:** Local flaps, oral cancer, physiotherapy

**To the Editor**:

We are humbled to read the letter from Dr Srivatsan Munusamy and colleagues in response to our paper titled “Local Flap Reconstructions in Oral Cavity Defects: An Insight from 104 Cases” published in July 2024 in the esteemed *Rambam Maimonides Medical Journal*.^1^

We sought to publish our experience reconstructing complex oral cancer resection defects using local flaps to highlight their versatility. Local flaps in the head and neck region can be a boon to surgeons when utilized in select cases. With this purpose in mind, our paper emphasized the long-term outcomes of local flaps in oral cancer patients. However, validating the functionality of the results using objective parameters seemed beyond the scope of our study.

Hence, we found the letter from Dr Munusamy and co-workers, titled “Optimizing Recovery in Oral Flap Surgeries: The Undervalued Role of Physiotherapy,” of great value and interest. The letter comprehensively addresses the true outcomes of a surgically treated oral cancer patient, mostly adjunct

with postoperative radiotherapy. These patients suffer from trismus, fibrosis of the neck musculature, and shoulder dysfunction that significantly impact the overall well-being of the patients. These issues not only affect the patients’ functional outcomes, such as speaking, eating, and day-to-day activities, but also affect their overall physical health and emotional stability. Hence, the role of physiotherapy is of paramount importance in helping these patients recover.

Dr Munusamy and colleagues have aptly highlighted the role of “prehabilitation” in the management of oral cancer patients. Early physical therapy intervention prepares the patients to navigate the long journey of oral cancer treatment. It not only aids in better post-treatment functional outcomes but also positively impacts the mental well-being of the patients. Furthermore, intensive postoperative physiotherapy should be advocated as early as when the patient is in the ward. This early intervention promises faster recovery with better mouth opening, improved oral intake, better speech, and less shoulder dysfunction.

Finally, we concur that a multi-disciplinary approach with active incorporation of a physiotherapist is the need of the hour to treat oral cancer patients. The structured physiotherapy regimens will drastically reduce the postoperative sequelae and morbidity in oral cancer patients. We firmly believe that surgeons should conduct more studies with objective assessment of the long-term functional outcomes of flap surgeries. These studies should incorporate physiotherapy regimens and grade the assessment of the recovery of patients. This will enhance the importance of prehabilitation and postoperative rehabilitation in oral cancer patients. In conclusion, the role of the physiotherapist, even though undervalued, is essentially one of the most crucial interventions that ensure a better quality of life for oral cancer patients.
